# Effects of the Treg/Th17 cell balance and their associated cytokines in patients with hepatitis B infection

**DOI:** 10.3892/etm.2014.2104

**Published:** 2014-12-03

**Authors:** YI CHEN, JIANKAI FANG, XUZHENG CHEN, CHEN PAN, XIAOLONG LIU, JINGFENG LIU

**Affiliations:** 1 The Liver Center of Fujian Province, Mengchao Hepatobiliary Hospital of Fujian Medical University, Fuzhou, Fuijan 350025, P.R. China; 2 Academy of Integrative Medicine, Fujian University of Traditional Chinese Medicine, Fuzhou, Fuijan 350108, P.R. China

**Keywords:** hepatitis B virus, T regulatory cell, T helper 17 cell, cytokine, interleukin

## Abstract

The extent to which T-cell-mediated immunity is impaired in patients with hepatitis B virus (HBV) infection remains controversial. In addition, the role of T regulatory (Treg) and T helper 17 (Th17) cells and their associated cytokines in immunity is not clear. In the present study, peripheral blood samples were collected from 44 patients with chronic hepatitis B virus, 14 asymptomatic hepatitis B carriers, 19 patients with liver cirrhosis and 20 healthy individuals. Flow cytometry was used to detect the percentages of T cell subsets in the samples, including CD3^+^, CD4^+^ and CD8^+^ T cells, Treg cells and Th17 cells. A cytometric bead array was conducted to detect the levels of interleukin (IL)-17, -6, -10 and -21, and transforming growth factor (TGF)-β. The data revealed that Treg cell levels decreased, while Th17 cell levels increased in the peripheral blood of HBV patients. As the extent of inflammation and fibrosis in the hepatic tissue increased, the frequency of Treg and Th17 cells in the peripheral blood did not significantly differ. In addition, the levels of Th17 cells were found to positively correlate with TGF-β and IL-21 levels. Therefore, analyzing the balance between Treg/Th17 cells and their associated cytokines may be a useful indicator in the diagnosis of HBV.

## Introduction

Hepatitis B is a serious health risk. The hepatitis B virus (HBV) is a non-cytopathogenic hepadnavirus. The prognosis of an HBV infection is closely associated with the condition of the immune system. The majority of liver damage in HBV patients is caused by the removal of infection from liver cells by the immune system, which produces a variety of inflammatory cytokines by non-specific T cells (1). Immunologically, the key to treating HBV is to restore an effective T cell response in patients with chronic hepatitis B (CHB) infection. Previous studies have confirmed that the immune system can remove cccDNA by a non-cellular destructive mechanism (1–3). Thus, the immunological treatment of HBV is attracting widespread attention and is expected to become an important approach for the treatment of CHB. However, there is currently no specific immune therapy that produces a good curative effect on HBV.

The functions of T regulatory (Treg) cells and T helper 17 (Th17) cells in the underlying mechanism of CHB are being increasingly investigated. However, the changes in Treg and Thl7 cell levels in the peripheral blood and the subsequent impact on the development of HBV infection have not yet been characterized, which is due primarily to the different definitions of target cells and detection methods. In addition, the interactions between associated cytokines remain unclear. Previous studies have demonstrated that chronic HBV infection increases the number of Treg cells in the peripheral blood and the liver, inhibiting HBV-specific T cell proliferation *in vitro* (4–6). Removing the Treg cells has been observed to enhance the specific immune reaction. Thus, the activity of CD4^+^CD25^+^ Treg cells in chronic HBV infection is one of the reasons for the persistent nature of HBV infection (7–9). Decreasing the number of Treg cells or inhibiting their activity has been hypothesized to overcome immune tolerance, strengthening the rate of virus removal. Previous studies on Treg cells in tumor-immunotherapy have demonstrated a significant effect on tumors through the use of mouse models (10–12). Prior to vaccine therapy, removing the Treg cells from kidney cancer patients has been shown to result in a 100-fold increase in specific T cell levels (13); however, research into the treatment of HBV infection using this method has not yet been reported. The aim of the present study was to investigate the changes in the levels of Treg and Th17 cells and their associated cytokines following HBV infection.

## Materials and methods

### Blood samples and instruments

Heparinized peripheral blood samples were obtained from patients with HBV in accordance with the CHB prevention guidelines (1). The use of human samples in the present study was approved by the Institution Review Board of Mengchao Hepatobilliary Hospital of Fujian Medical University (Fizhou, China). Written informed consent was received from all participants. Clinical diagnosis of patients with HBV met the diagnostic criteria for chronic hepatatis of the Chinese Association for the study of Liver Diseases (WS299-2008). There were four blood sample groups: Liver cirrhosis (LC), CHB, asymptomatic carrier (ASC) and normal control (NC). Clinical characteristics were recorded (Table I) and analyzed using the paired Student’s t test.

A FASCabilur flow cytometer (BD Biosciences, Franklin Lakes, NJ, USA) was used to measure the levels of Treg cells, Th17 cells and cytokines, including TGF-β, IL-17, -6, -10 and -21, in the peripheral blood. In 83/90 patients with HBV, cytokines were detected using a cytometric bead array (CBA). The remaining seven patients were not included as their serum samples were not collected. In addition, peripheral blood samples were obtained from 20 healthy controls for measurement of cytokines, and Treg/Th17 levels.

### T cell subsets

Fluorescence-activated cell sorting (FACS) analysis was conducted to measure the levels of CD3^+^, CD4^+^ and CD8^+^ T cells in the peripheral blood of the four groups, and subsequently the CD4^+^/CD8^+^ T cell ratio. Measurements were collected using Multiset software (BD Biosciences), which is a four-color lymphocyte automated analysis.

### Treg levels

FACS analysis was conducted to measure the Treg cell frequency in the peripheral blood. The levels were measured using CD4 PerCP-CY5.5, CD25 PE and CD127 Alexa Fluor 647 antibodies (0.05 mg/ml BD Biosciences). The cells were incubated with the antibodies for 15 min prior to abstersion. CD4^+^CD25^−^CD127^+^ cells were used as surface markers to indicate a positive cell group.

### Th17 levels

Th17 cell levels were measured using IL-17 PE, mouse IgG1 PE, CD8 FITC and CD3 PerCP antibodies (BD Biosciences). Blood samples were stimulated using phorbol 12-myristate 13-acetate (50 ng/ml; Sigma-Aldrich, St. Louis, MO, USA) and ionomycin (1 μg/ml; Sigma-Aldrich), and incubated for 5 h at 37°C. The CD8 FITC and CD3 PerCP antibodies were used as surface markers for staining. Fixation and permeabilization of the cells was performed using an intracellular staining kit (0.05 mg/ml; BD Biosciences), according to manufacturer’s instructions. IL-17 PE was an intracellular antibody and mouse IgG1 PE was used as the negative control.

### Cytokine levels

Multi-target streaming protein quantitative technology (BD-Pharmigen Cytometric Bead Array; BD Biosciences) was used to detect the cytokine levels in the peripheral blood, following the manufacturer’s instructions.

### Statistical analysis

All statistical analyses were performed using SPSS version 19.0 (SPSS Inc., Chicago, IL, USA). Quantitative data between two groups were compared by two-tailed unpaired student’s t test, and the data were expressed as the mean ± standard deviation. P<0.05 was considered to indicate a statistically significant difference.

## Results

### Percentages of T cell subsets in the peripheral blood of the four groups show no significant differences

**Figure 1:**
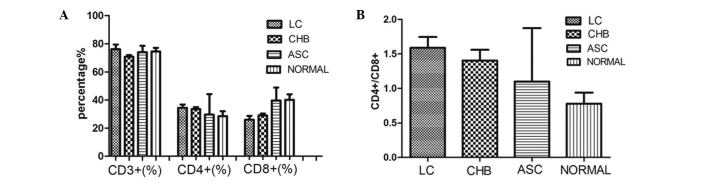
(A) Percentages of CD3^+^, CD4^+^ and CD8^+^ T cells and the (B) ratio of CD4^+^/CD8^+^ T cells in the peripheral blood of the four groups (P>0.05). LC, liver cirrhosis; CHB, chronic hepatitis B; ASC, asymptomatic carrier; NC, normal control.

Percentages of T cells in the peripheral blood can be used as an indication of the functional status of the immune system. To analyze the functional status of the immune system in the four groups, the levels of CD3^+^, CD4^+,^ and CD8^+^ T cells, and the CD4^+^/CD8^+^ T cell ratio, were analyzed using flow cytometry (Fig. 1). The percentages of CD3^+^ T cells in the LC, CHB, ASC and NC groups were 76.19±15.40, 70.80±7.69, 74.00±7.81 and 74.50±6.47%, respectively (Table II). The percentages of CD4^+^ T cells in the LC, CHB, ASC and NC groups were 34.48±11.03, 33.64±8.76, 29.67±25.15 and 28.50±8.64%, respectively. Furthermore, the percentages of CD8^+^ T cells in the LC, CHB, ASC and NC groups were 26.00±12.28, 29.07±8.87, 39.67±15.95 and 40.17±9.70%, respectively. No statistically significant differences were observed in the percentages of T cell subsets (CD3^+^, CD4^+^ and CD8^+^ T cells) among the four groups (P>0.05). In addition, the ratios of CD4^+^/CD8^+^ cells in the LC, CHB, ASC and NC groups were 1.58±0.70, 1.40±1.01, 1.10±1.33 and 0.78±0.38, respectively. There were no statistically significant differences among the four groups with regard to the CD4^+^/CD8^+^ ratios (P>0.05).

### Percentages of Treg and Th17 cells are elevated in the LC, CHB and ASC groups

**Figure 2:**
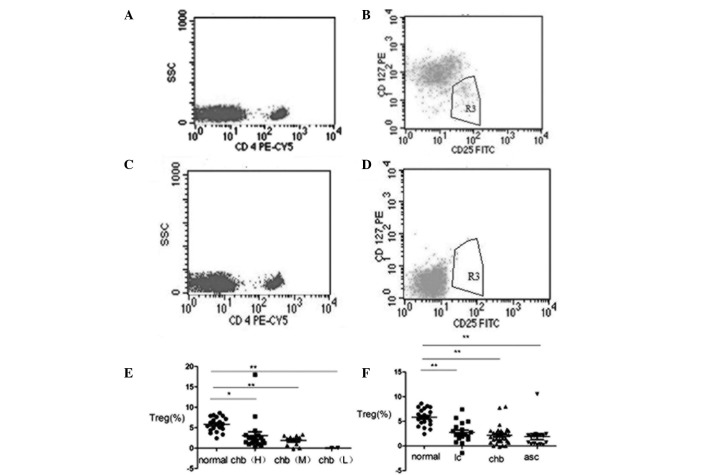
Flow cytometric analysis of the Treg cell levels. The percentages in each image represent the levels of the analyzed cell subsets, (A and B) CD4^+^CD25^+^CD127(low) and (C and D) negative control (immunoglobulin G antibody). Percentages of Treg cells (E) in the heavy, middle and low CHB groups and (F) in the CHB, LC and ASC groups. ^*^P<0.05 and ^**^P<0.01. Treg, T regulatory; CHB, chronic hepatitis B; LC, liver cirrhosis; ASC, asymptomatic carrier.

**Figure 3:**
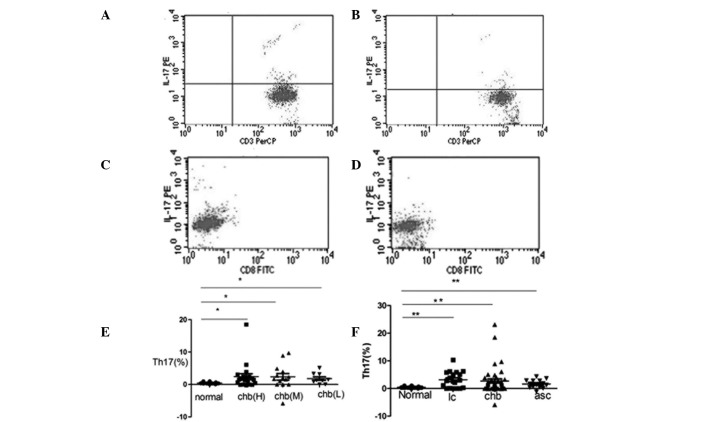
Flow cytometric analysis of IL-17^+^ Th17 cells. The percentages in each figure represent the levels of the analyzed cell subsets. (A and C) Blood samples were stimulated with phorbol 12-myristate 13-acetate and ionomycin. (B and D) Blood samples were not stimulated. (E) Percentages of Th17 cells in the heavy, middle and low CHB groups and the normal control. (F) Percentages of Th17 cells in the normal, LC, CHB and ASC groups. ^*^P<0.05 and ^**^P<0.01. IL, interleukin; Th17, T helper 17; CHB, chronic hepatitis B; LC, liver cirrhosis; ASC, asymptomatic carrier.

Levels of CD4^+^CD25^+^CD127^−^ Treg cells and CD4^+^IL-17^+^ Th17 cells were analyzed and the representative flow cytometry results are shown in Figs. 2 and 3. Furthermore, the percentages of Treg and Th17 cells in patients with different stages of CHB (Figs. 2E and 3E) were analyzed. The percentages of CD4^+^CD25^+^CD127^−^ Treg cells in the NC group and heavy, middle and light CHB groups were 5.82±1.76, 3.08±3.96, 1.89±1.26 and 0.025±0.04%, respectively. The percentages of Th17 cells in the NC group and heavy, middle and light CHB groups were 0.44±0.24, 2.38±4.10, 2.36±3.85 and 1.82±1.69, respectively (Table III). There were no statistically significant differences observed among the heavy, middle and light CHB groups (P>0.05).

Percentages of Treg and Th17 cells in the peripheral blood of the NC, LC, CHB and ASC groups were also analyzed by flow cytometry (Figs. 2F and 3F). The percentages of Treg cells in the NC, LC, CHB and ASC groups were 5.83±1.76, 2.74±2.06, 2.17±1.88 and 1.90±2.45%, respectively. Statistically significant differences were observed between the NC group and the other three groups (P<0.05, P<0.01). The percentages of Th17 cells in the NC, LC, CHB and ASC groups were 0.44±0.24, 3.19±2.94, 2.72±4.79 and 1.63±1.53%, respectively (Table II). Similarly, statistically significant differences were identified between the NC group and the other three groups (P<0.01).

### Cytokine levels in the peripheral blood of the NC group are significantly different compared with the other groups

**Figure 4:**
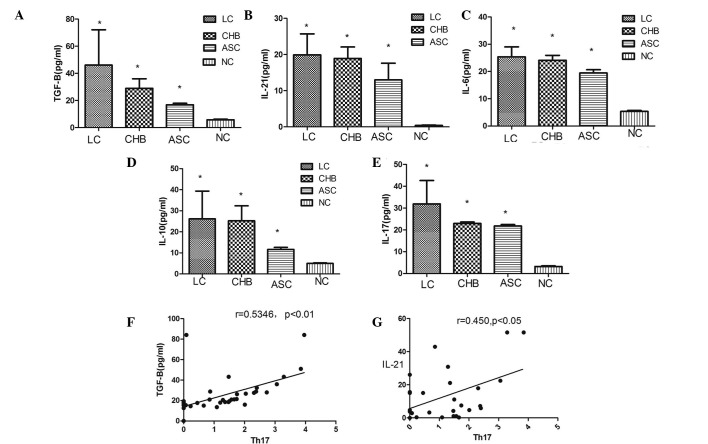
Expression levels of (A) TGF-β, (B) IL-21, (C) IL-6, (D) IL-10 and (E) IL-17 were measured using a cytometric bead assay. Correlation analyses were conducted between the levels of Th17 cells and (F) TGF-β and (G) IL-21 levels in the CHB group, using Spearman’s rank correlation. *P<0.05, vs the NC group. TGF-β, transforming growth factor-β; IL, interleukin; LC, liver cirrhosis; CHB, chronic hepatitis B; ASC, asymptomatic carrier; NC, normal control; Th17, T helper 17.

CBAs were performed to measure the expression levels of the cytokines, TGF-β, IL-21, -6, -10 and -17, in the peripheral blood of the four groups (Fig. 4). Cytokine levels in the NC group differed significantly when compared with the other three groups. In addition, Th17 cell levels were shown to positively correlate with the TGF-β and IL-21 levels.

## Discussion

Previous studies have shown that the CD4^+^ T cell subset includes Th1, Th2, Treg and Th17 cells (14,15). Under specific conditions, CD4^+^ T cells can differentiate into these cell types and secrete different cytokines to mediate an immune response. For example, under the induction of TGF-β, CD4^+^ T cells may differentiate into Treg cells, which secrete TGF-β and Foxp3. Under the induction of TGF-β and IL-6, CD4^+^ T cells may differentiate into Th17 cells, producing IL-17, -21 and -23, amongst other cytokines (16).

Treg and Th17 cells are closely associated with a variety of autoimmune diseases. These cells are involved not only in the regulation of immune tolerance, but also play an important role in tumor immunity (17–20). Treg cells are a type of T lymphocyte with unique functions. Treg cells have been intensely studied due to their capacity to secrete IL-4, IL-10 and TGF-β. Th17 cells primarily secrete IL-17, and have numerous immune response-associated functions, including promoting the proliferation, maturation and chemotactic activity of neutrophils. In addition, Th17 cells may costimulate the activation of T cells and promote the maturation of dendritic cells (21).

In the present study, the T cell immunity of HBV patients was systematically investigated by analyzing the balance between Treg/Th17 cells and their associated cytokines. Peripheral blood samples were collected from four groups of individuals, including 44 patients with CHB, 14 ASCs, 19 patients with LC and 20 healthy controls. Samples were stained with different cell surface markers and intracellular makers prior to cytometric analysis. Multi-target streaming protein quantitative technology (CBA) was used to measure the cytokine levels, with the aim of providing further data for improving the treatment of HBV.

Firstly, the total percentages of peripheral CD3^+^, CD4^+^ and CD8^+^ T cells were measured. The results showed that there were no significant differences among the four groups, with regard to the total percentage of CD3^+^, CD4^+^ and CD8^+^, as compared with the NC group.

Secondly, the percentages of Treg and Th17 cells were measured. The results indicated that the levels of Treg cells in the peripheral blood of HBV patients decreased, however, the levels of Th17 cells increased. As the extent of inflammation and fibrosis in the hepatic tissue increased, the levels of Treg and Th17 cells in the peripheral blood samples exhibited heavy, middle and low distribution (Figs. 2E and 3E), in spite of there being no statistically significant differences in their distribution. The percentages of CD4^+^CD25^+^CD127^−^ Treg cells decreased significantly in the three CHB groups when compared with the NC group (Fig. 2F). Furthermore, the levels of CD4^+^IL-17^+^ Th17 cells in the three CHB groups increased significantly when compared with the NC group (Fig. 3F). The Treg cell results obtained in the present study differ from those obtained in previous studies (13,22). Yu *et al* (23) found that levels of Treg cells were significantly increased in CHB patients (n=70) and slightly increased in patients with HBV/LC (n=28) when compared with normal controls (n=20), whereas Th17 cell levels were markedly increased in the CHB and LC patients. In the study by Zhang *et al* (24), samples were collected from 14 patients at various stages of HBV-related acute-on-chronic liver failure (ACLF), and were compared with 30 CHB patients and 18 healthy controls. The frequency of circulating Treg cells was significantly lower in the patients at the remission stage of ACLF when compared with those in the progression stage or in the CHB group. The increase in Th17 cells and concomitant decrease in Treg cells created an imbalance in the remission stage ACLF patients, which negatively correlated with disease progression. The results of the present study concur with these findings. However, future studies are required to further the understanding into the balance of Treg/Th17 cells in HBV patients.

Cytokines are important mediators of the immune response, including the immune response in the liver cells of HBV patients. In addition to being secreted by immune cells, cytokines exert feedback effects on the immune cells. During the course of HBV infection, the functions of a number of cytokines are ambiguous. For example, TGF-β may stimulate CD4^+^ T cells to remove HBV by activating Treg cells. Alternatively, TGF-β, IL-6 and IL-21 may promote the development of Th17 cells, which can enhance the local immune response *in vivo* (25–27). In the present study, the levels of TGF-β, IL-21, -6, -10 and -17 were detected using a CBA. Th17 cell levels were found to positively correlate with the levels of TGF-β and IL-21, indicating that TGF-β and IL-21 are key cytokines in the determination of cell differentiation (Fig. 4F and G). TGF-β stimulation can result in the production of Treg or Th17 cells. As an autocrine regulatory factor, IL-21 induces differentiation and inhibits Thl7 and Treg cell function. IL-21 function was revealed to be similar to that of IL-6. Therefore, TGF-β and IL-21 may influence T cells that can be induced to differentiate into Thl7 cells. Increasing levels of these cytokines may reduce immune function and promote HBV development by affecting the Th17/Treg cell ratio. Thus, an imbalance in these cell types may be associated with the promotion and development of HBV infection.

## Figures and Tables

**Table I:** Patient characteristics.

Patient characteristics	CHB (n=44)	ASC (n=14)	LC (n=19)	Normal (n=20)
Gender (male:female), n	33:11	10:3	14:5	10:10
Median age, years (range)	35 (9–63)	33 (15–59)	67 (29–76)	32 (23–45)
Grade (low:middle:heavy), n	20:15:9			
Other diseases (HDV), n	2			

Direct comparison of the clinical features of patients in the CHB, ASC, LC and normal groups. CHB, chronic hepatitis B; ASC, asymptomatic carrier; LC, liver cirrhosis; HDV, hepatitis D virus.

**Table II:** Proportion of T lymphocytes in the four groups (%).

Cell types	LC	CHB	ASC	Normal
CD3^+^ T cell	76.19±15.40	70.80±7.69	74.00±7.81	74.50±6.47
CD4^+^ T cell	34.48±11.03	33.64±8.76	29.67±25.15	28.50±8.64
CD8^+^ T cell	26.00±12.28	29.07±8.87	39.67±15.95	40.17±9.70
CD4^+^/CD8^+^ ratio	1.58±0.70	1.40±1.01	1.10±1.33	0.78±0.38
CD4^+^CD25^+^ Treg	2.74±2.06^a^	2.17±1.88^a^	1.90±2.45^a^	5.83±1.76^a^
CD4^+^IL17^+^ Th17	3.19±2.94^a^	2.72 ±4.79^a^	1.63±1.53^a^	0.44±0.24^a^

Quantitative results from flow cytometric analysis.

a P<0.01, vs. normal group.

LC, liver cirrhosis; CHB, chronic hepatitis B; ASC, asymptomatic hepatitis B carrier; IL-17, interleukin-17; Treg, T regulatory; Th17, T helper 17.

**Table III:** Proportions of T lymphocytes in the three CHB groups (%).

Cell types	Heavy group	Middle group	Mild group	Normal group
CD4^+^CD25^+^ Treg	3.08±3.96^a^	1.89±1.26^b^	0.025±0.035^b^	5.82±1.76
CD4^+^IL17^+^ Th17	2.38±4.10^b^	2.36±3.85^b^	1.82±1.69^b^	0.44±0.24

Quantitative results from flow cytometric analysis.

a P<0.05 and

b P<0.01, vs. normal group.

CHB, chronic hepatitis B; Treg, T regulatory; IL-17, interleukin-17; Th17, T helper 17.

## Abbreviations

LC: liver cirrhosis
HBV: hepatitis B virus
CHB: chronic hepatitis B virus
ASC: asymptomatic carrier
NC: normal control
CBA: cytometric bead array
IL: interleukin
Treg: T regulatory
Th17: T helper 17
TGF-β: transforming growth factor-β
FACS: fluorescence-activated cell sorting
ACLF: acute-on-chronic liver failure
